# Illness in Long-Term Travelers Visiting GeoSentinel Clinics

**DOI:** 10.3201/eid1511.090945

**Published:** 2009-11

**Authors:** Lin H. Chen, Mary E. Wilson, Xiaohong Davis, Louis Loutan, Eli Schwartz, Jay Keystone, Devon Hale, Poh Lian Lim, Anne McCarthy, Effrossyni Gkrania-Klotsas, Patricia Schlagenhauf, for the GeoSentinel Surveillance Network

**Affiliations:** Harvard University, Boston, Massachusetts, USA (L.H. Chen, M.E. Wilson); Mount Auburn Hospital, Cambridge, Massachusetts, USA (L.H. Chen, M.E. Wilson); Centers for Disease Prevention and Control, Atlanta, Georgia, USA (X. Davis); University of Geneva, Geneva, Switzerland (L. Loutan); Chaim Sheba Medical Centre, Tel Hashomer, Israel (E. Schwartz); Toronto General Hospital, Toronto, Ontario, Canada (J. Keystone); University of Utah, Salt Lake City, Utah, USA (D. Hale); Tan Tock Seng Hospital, Singapore (P.L. Lim); University of Ottawa, Ottawa, Ontario, Canada (A. McCarthy); University of Cambridge, Cambridge, UK (E. Gkrania-Klotsas); University of Zürich, Zürich, Switzerland (P. Schlagenhauf); 1 Additional members of the GeoSentinel Surveillance Network who contributed data are listed at the end of this article.

**Keywords:** Long-term travelers, missionaries, business travelers, special travel populations, bacteria, viruses, research

## Abstract

Length of travel appears to be associated with health risks.

Length of travel appears to be associated with health risks. GeoSentinel Surveillance Network data for 4,039 long-term travelers (trip duration >6 months) seen after travel during June 1, 1996, through December 31, 2008, were compared with data for 24,807 short-term travelers (trip duration <1 month). Long-term travelers traveled more often than short-term travelers for volunteer activities (39.7% vs. 7.0%) and business (25.2% vs. 13.8%). More long-term travelers were men (57.2% vs. 50.1%) and expatriates (54.0% vs. 8.9%); most had pretravel medical advice (70.3% vs. 48.9%). Per 1,000 travelers, long-term travelers more often experienced chronic diarrhea, giardiasis, *Plasmodium falciparum* and *P. vivax* malaria, irritable bowel syndrome (postinfectious), fatigue >1 month, eosinophilia, cutaneous leishmaniasis, schistosomiasis, and *Entamoeba histolytica* diarrhea. Areas of concern for long-term travelers were vector-borne diseases, contact-transmitted diseases, and psychological problems. Our results can help prioritize screening for and diagnosis of illness in long-term travelers and provide evidence-based pretravel advice.

Travelers have many reasons for long durations of travel, including diplomatic work, education and research, missionary work, Peace Corps volunteer (PCV) work, military operations, backpacking trips, and corporate expatriate assignments (*1**–**7*). Longer trips often are assumed to be associated with increased risk for some health problems, but few studies have compared the types and causes of illness in travelers on the basis of duration of travel. Previous studies suggested that long-term travelers are more likely than short-term travelers to acquire malaria (*8*) and that recommendations should be tailored individually (*9*). Other illness also might be more common in long-term than in short-term travelers.

To evaluate the effect of trip duration on illness, we compared illnesses by duration of travel for travelers seeking treatment at GeoSentinel Surveillance Network sites. We also characterized long-term travelers’ demographics, travel patterns, and travel-related illnesses.

## Methods

GeoSentinel Surveillance Network (www.istm.org/geosentinel/main.html) sites are clinics on 6 continents that specialize in travel or tropical medicine and contribute data on travel-related illnesses and trip information. Our study comprised data from ill travelers visiting GeoSentinel sites from June 1, 1996, through December 31, 2008.

### Inclusion Criteria

**Figure 1:**
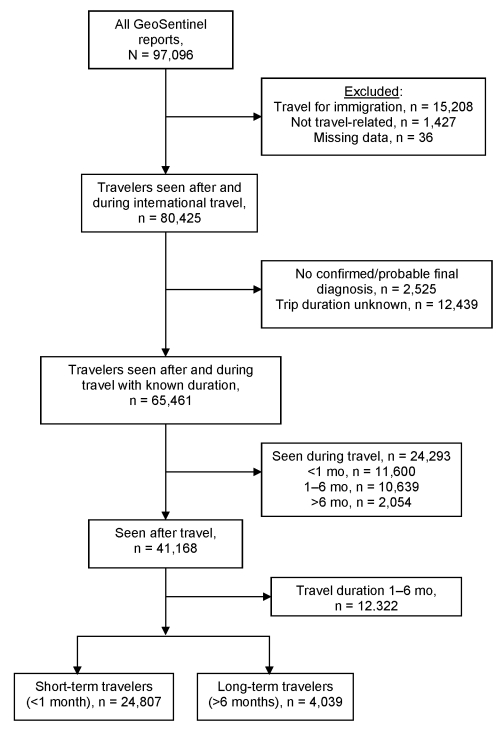
Flow chart for analysis of illness and injury in long-term travelers, GeoSentinel Surveillance Network, June 1996–December 2008.

Persons in our study must have crossed an international border within the past 10 years and then sought treatment or medical advice at a GeoSentinel site for a presumed travel-related illness. Only travelers with confirmed and probable diagnoses (including a healthy screening result) were included (Figure 1), and >1 diagnosis per patient was possible. Final diagnoses were assigned by a clinician.

Data were collected according to a standardized, anonymous questionnaire and entered into a Structured Query Language database. The questionnaire comprised demographic data (i.e., age, gender, country of birth, country of residence), travel history, inpatient or outpatient status, major diagnoses, pretravel encounter for travel health advice, reason for most recent travel, patient classification, and risk level qualifier (e.g., prearranged or organized travel, risk travel, and expatriate status). Included in the analysis were persons traveling for tourism, visits to friends and relatives (VFR), business, military purposes, education, research, or missionary/volunteer work. We excluded records lacking an exposure destination or duration of travel, as well as records of immigrating travelers, travelers with multiple trips without a specified location of exposure, final diagnoses attributed to travel of unspecified duration, travel duration of 1–6 months, and travelers seen during travel. We defined long-term travel as duration >6 months and short-term travel as duration <1 month. Data for specific diagnoses and syndromes were analyzed for travelers seen after travel.

### Diagnostic Categories

Final diagnoses were assigned a diagnostic code from a standardized list of ≈500 diagnoses, which were categorized into 21 broad syndrome groups, as previously described (*10*). Diagnosis codes with clear causal routes were analyzed by the following categories: ingestion, vector-borne, contact (including respiratory, droplets, blood, body fluid, sexual transmission), environment (water, soil, animal contact), psychosocial, and medication intolerance.

### Statistical Analysis

Data were analyzed by using SAS software, version 9 (SAS Institute, Cary, NC, USA). Proportionate illness was calculated as the number of patients with a specific or grouped diagnosis as a proportion of short-term or long-term travelers, expressed per 1,000 persons in that category (*10**,**11*). Statistical significance was determined by using χ^2^ tests for categoric variables. For the most common diagnoses in long-term travelers, odds ratios (ORs) with 95% confidence intervals (CIs) were used to compare long-term with short-term travelers. A 2-sided significance level of p<0.05 was chosen. To avoid a regional bias (i.e., some exposure regions differed significantly between long-term and short-term travelers), we calculated ORs for the most common diagnoses in long-term travelers for the specific regions.

For long-term travelers, we performed multivariate logistic regressions to identify significant factors associated with various diseases. We adjusted for age, sex, pretravel encounters, reason for travel, and geographic region visited. Significant factors (p<0.05) were determined from stepwise selection.

## Results

### Demographics

Of 41,168 eligible persons seen after travel, 24,807 (60.3%) traveled for <1 month (short-term travelers), 12,322 (29.9%) traveled for 1–6 months, and 4,039 (9.8%) traveled for >6 months (long-term travelers). Mean ages were 33 years for long-term travelers and 38 for short-term travelers (Appendix Table 1). The male:female ratio was 4:3. Most long-term travelers (90%) were 20–64 years of age, and most originated from countries in western Europe (43%) or North America (29%). Median duration for long-term travel was 365 days (mean 693 days, range 243–713 days) and for short-term travel was 14 days (mean 15 days, range 9–21 days).

Long-term travelers more often traveled for volunteer activities or research (40% vs. 7%) and business (25% vs. 14%) and less often for tourism (29% vs. 70%). A larger proportion of long-term than short-term travelers were male (57% vs. 50%) and expatriates (54% vs. 9%), and most had sought pretravel medical advice (70% vs. 49%).

Long-term travelers more often traveled to sub-Saharan Africa (34%) and South America (16%) than did short-term travelers. Similar proportions of long- and short-term travelers went to south-central Asia (14% and 13%, respectively), and the proportion of long-term travelers with exposure in Southeast Asia was lower than that of short-term travelers. Intervals between return from travel to visit to a GeoSentinel site after long-term travel were <1 week (32%), 1–6 weeks (38%), and >6 weeks (30%).

### Syndromes

Predominant syndromes in long-term travelers seen after travel were febrile systemic illness, acute diarrheal syndrome, dermatologic problems, and other gastrointestinal problems ( Appendix Table 2). A larger proportion of long-term than short-term travelers were determined to be healthy (196/1,000 travelers vs. 49/1,000 travelers).

### Most Common Diagnoses and Proportionate Illness

**Figure 2:**
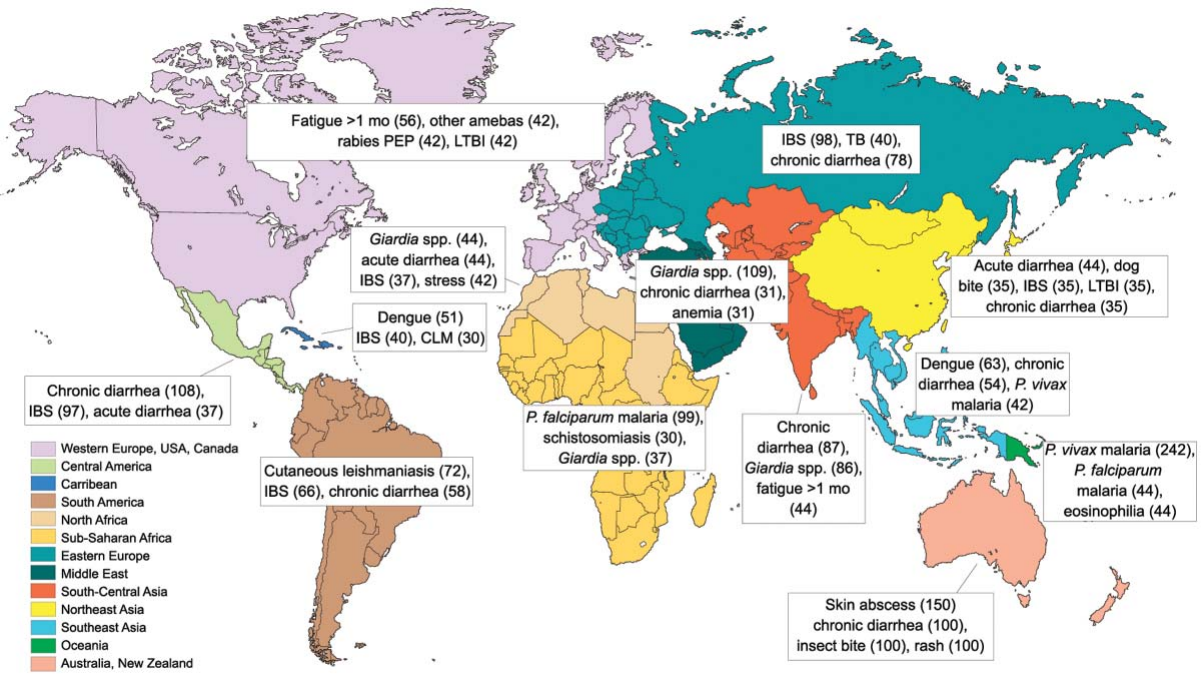
Proportionate illness (per 1,000 ill travelers) for the most frequent diagnoses in long-term travelers, by world geographic region visited, GeoSentinel Surveillance Network, June 1996–December 2008. PEP, postexposure prophylaxis; IBS, irritable bowel syndrome; TB, tuberculosis; LTBI, latent TB infection; CLM, cutaneous larva migrans; *P., Plasmodium*.

Proportions of common diagnoses in long-term travelers by world region visited are shown in Figure 2. Long-term travelers were significantly more likely than short-term travelers to have chronic diarrhea (OR 1.20, 95% CI 1.04–1.38); giardiasis (OR 1.57, 95% CI 1.32–1.86); *P. falciparum* malaria (OR 1.5, 95% CI 1.26–1.78); irritable bowel syndrome (postinfectious) (OR 1.69, 95% CI 1.41–2.01), *P. vivax* malaria (OR 2.46, 95% CI 1.92–3.17); fatigue >1 month (OR 3.09, 95% CI 2.86–4.01); eosinophilia (OR 3.34, 95% CI 2.53–4.42); cutaneous leishmaniasis (OR 4.89, 95% CI 3.55–6.73); unspecified schistosomiasis (OR 4.45, 95% CI 3.16–6.25 [OR 4.26 for all schistosomiasis diagnoses together]); and *Entamoeba histolytica* diarrhea (OR 3.33, 95% CI 2.34–4.73) (Table 1). The most frequent regions of exposure for long-term versus short-term travelers were sub-Saharan Africa (34.26% vs. 24.59%; OR 1.60, 95% CI 1.48–1.73), followed by South America (16.38% vs. 7.30%; OR 2.49, 95% CI 2.24–2.76) and Southeast Asia (12.59% vs. 18.90%; OR 0.56, 95% CI 0.5–0.62, p = 0.00000) (Appendix Table 1). Diagnoses of acute infections (such as dengue fever, rickettsiosis, acute diarrhea, acute bacterial diarrhea, influenza, and sexually transmitted infections), animal bites, and insect bites were significantly more common in short-term travelers.

**Table 1:** Most common diagnoses for long-term travelers (n = 4,742) seen after travel and proportionate illness compared with short-term travelers (n = 28,618), GeoSentinel Surveillance Network, June 1996–December 2008*

Rank†	Diagnosis	Rate/1,000 travelers	Odds ratio (95% confidence interval)				
			Overall	South America	Southeast Asia	Sub-Saharan Africa	All other regions
1	Diarrhea, chronic unknown	50	1.20‡ (1.04–1.38)	1.19 (0.81–1.75)	1.59§ (1.05–2.39)	0.92 (0.64–1.31)	1.19 (0.99–1.44)
2	*Giardia* spp.	36	1.57‡ (1.32–1.86)	0.85 (0.49–1.47)	1.48 (0.80–2.73)	1.93‡ (1.39–2.67)	1.59‡ (1.26–2.01)
3	Irritable bowel syndrome, postinfectious	36	1.69‡ (1.41–2.01)	2.13§ (1.42–3.18)	2.76§ (1.52–5.02)	1.59¶ (1.03–2.45)	1.42§ (1.11–1.80)
4	Malaria, *Plasmodium falciparum*	36	1.50‡ (1.26–1.78)	NA	5.05‡ (2.58–9.88)	1.05 (0.86–1.27)	2.77§ (1.58–4.87)
6	Malaria, *P. vivax*	19	2.46‡ (1.92–3.17)	0.83 (0.33–2.07)	4.79‡ (2.86–8.01)	1.14 (0.67–1.94)	3.66‡ (2.57–5.22)
8	Fatigue >1 month (not febrile)	18	3.09‡ (2.86–4.01)	3.45§ (1.59–7.50)	1.94 (0.86–4.37)	1.79 (0.98–3.25)	4.27‡ (3.01–6.05)
9	Eosinophilia	17	3.34‡ (2.53–4.42)	3.49§ (1.56–7.83)	3.11§ (1.46–6.60)	4.11‡ (2.46–6.84)	2.89‡ (1.91–4.37)
11	Leishmaniasis, cutaneous	14	4.89‡ (3.55–6.73)	9.14‡ (5.15–16.24)	NA	0.77 (0.09–6.40)	2.30§ (1.35–3.92)
12	Schistosomiasis, human species unknown#	13	4.45‡ (3.16–6.25)	2.92 (0.18–46.68)	3.30 (0.34–31.80)	3.10‡ (2.09–4.59)	7.44‡ (3.83–14.47)
17	TB, positive PPD or IGRA	11	3.26‡ (2.33–4.56)	2.92 (0.73–11.72)	24.27‡ (8.52–69.17)	2.44¶ (1.13–5.26)	2.68‡ (1.71–4.18)
18	*Entamoeba histolytica*, diarrhea	11	3.33‡ (2.34–4.73)	2.57 (0.93–7.10)	1.52 (0.34–6.77)	3.88‡ (1.95–7.72)	3.52‡ (2.23–5.56)
21	Stress	9	5.70‡ (3.77–8.61)	NA	1.65 (0.20–13.73)	7.57‡ (3.13–18.30)	5.55‡ (3.32–9.30)
22	Epstein-Barr virus	8	2.60‡ (1.72–3.91)	12.86‡ (3.65–45.27)	2.99¶ (1.20–7.48)	0.38 (0.05–2.96)	2.29§ (1.30–4.03)
25	Strongyloidiasis, simple intestinal	7	1.85§ (1.24–2.75)	0.83 (0.17–4.01)	3.11¶ (1.14–8.53)	1.62 (0.88–2.99)	1.89 (0.94–3.81)

*Long-term travel is defined as >6 mo, short-term travel as <1 mo. NA, not applicable; TB, tuberculosis; PPD, purified protein derivative test; IGRA, interferon-gamma release assay. †Among 25 most common illnesses for all travelers. ‡p<0.0001. §p<0.01. ¶p<0.05. #Aggregated schistosomiasis diagnoses (mansoni, haematobium, japonicum, mekongi, and unknown) are grouped together and shown in Appendix Table 3,.

### Comparison of Diagnoses by Causal Route

Long-term travelers most commonly had diagnoses related to diseases with transmission by vectors or by ingestion. Larger proportions of long-term than short-term travelers had vector-borne diseases, contact-transmitted diseases (person-to-person, droplet, respiratory, sexually transmitted), and psychological problems (Table 2). Diagnoses for long-term travelers varied for travel and region of exposure ( Appendix Table 2).

**Table 2:** Comparison of rates for diagnoses among long-term and short-term travelers seen after travel by causal routes and preventive measures, GeoSentinel Surveillance Network, June 1996–December 2008*†

Grouped diagnoses	Rate/1,000 travelers		Odds ratio (95% CI)
	Short-term travelers	Long-term travelers	
Vector-borne infections	76	109	1.47 (1.33–1.63)
Dengue	24	17	0.69 (0.551–0.88)
Chikungunya	2	2	1.16 (0.59–2.29)
Leishmaniasis	3	14	4.89 (3.55–6.73)
Malaria, all species	39	68	1.83 (1.61–2.08)
Rickettsiosis	8	2	0.22 (0.11–0.45)
Filariasis	2	5	3.22 (1.981–5.24)
Ingestion	257	219	0.81 (0.75–0.87)
Enteric fever	5	9	1.70 (1.20–2.41)
Hepatitis A	2	3	1.21 (0.67–2.19)
Diarrhea, acute	123	41	0.31 (0.27–0.36)
Diarrhea, chronic	45	54	1.20 (1.04–1.38)
GI bacteria	34	15	0.42 (0.33–0.53)
Giardiasis	24	36	1.57 (1.32–1.86)
GI parasites	55	108	2.08 (1.88–2.312)
Contact‡	33	38	1.15 (0.70–1.90)
Influenza	8	5	0.60 (0.39–0.92)
Latent TB (positive PPD or IGRAs)	4	11	3.26 (2.33–4.56)
Acute mononucleosis syndrome (CMV, EBV, other)	7	11	1.60 (1.18–2.18)
Hepatitis B	2	2	0.67 (0.31–1.47)
Hepatitis C	1	2	1.73 (0.85–3.49)
Other sexually transmitted infections	7	4	0.67 (0.43–1.05)
HIV (acute infection)	2	1	0.39 (0.12–1.27)
Environment	119	87	0.71 (0.63–0.79)
Schistosomiasis	6	24	4.26 (3.35–5.42)
Strongyloides	4	7	1.85 (1.24–2.75)
Hookworm	2	2	1.26 (0.62–2.60)
Animal bite	44	13	0.28 (0.22–0.37)
Other skin contact, noninfectious	60	18	0.29 (0.23–0.36)
Fungal infection (superficial/cutaneous mycosis)	4	10	2.33 (1.66–3.28)
Rash	19	19	0.98 (0.78–1.23)
Psychological	15	40	2.80 (2.35–3.33)
Anxiety	3	4	1.60 (0.96–2.65)
Depression	2	6	3.03 (1.89–4.86)
Psychosis, nonmefloquine	1	2	3.89 (1.68–8.99)
Stress	2	9	5.70 (3.77–8.61)
Fatigue >1 mo	6	18	3.09 (2.86–4.01)
Adverse events from medication or vaccine	7	3	0.44 (0.26–0.74)
Mefloquine intolerance	4	1	0.19 (0.07–0.52)
Medication intolerance, nonmefloquine	3	2	0.83 (0.44–1.56)

*Long-term travel defined as >6 mo, short-term as <1 mo. CI, confidence interval; GI, gastrointestinal; TB, tuberculosis; PPD, purified protein derivative; IGRA, interferon-gamma release assay; CMV, cytomegalovirus; EBV, Epstein-Barr virus. †Diagnoses with proportionate illness <1/1,000 are omitted from table listing, such as hepatitis E, hepatitis delta, meningococcal meningitis, *Haemophilus influenzae* type b, pneumonia, pneumococcal pneumonia, varicella, chronic hepatitis, leptospirosis, altitude sickness, posttraumatic stress disorder, substance abuse, insomnia, delusional parasitosis, trauma, and violence exposure. Some patients may have >1 diagnosis. ‡Includes respiratory illnesses, blood/body fluid exchange, and sexually transmitted infections.

## Discussion

Existing data are limited regarding the number and proportion of all long-term travelers. This analysis of the GeoSentinel Surveillance Network found that long-term travelers constituted 9.8% of all travelers visiting GeoSentinel sites. In comparison, 5 travel medicine clinics in the Boston area found that ≈5% of travelers planned trips of ≥4 months’ duration (*12*). More than 66% of long-term travelers seen in the GeoSentinel Network had pretravel encounters, a higher percentage than shown in airport surveys of all travelers (range 31%–52% [*13**–**15*]). Many organizations, such as missions, corporations, and aid agencies, require health screening of their employees or participants after long-term overseas service, which may have resulted in the high yield of healthy diagnoses (196/1,000 travelers). Particular areas to consider for pretravel counsel for long-term travelers are vector-borne and contact-transmitted diseases and psychological problems.

### Ingestion Transmission

In our analysis, ingestion was the most common attributable route of transmission for diseases in long-term travelers, although long-term travelers sought treatment less frequently than short-term travelers for ingestion-transmitted diseases (OR 0.81, p<.0001). Enteric fever, acute diarrhea, chronic diarrhea, giardiasis, and other gastrointestinal parasites were reported significantly more often in long-term than short-term travelers (p = 0.0024 for enteric fever, p<0.0001 for the rest). Young age was associated with giardiasis and other gastrointestinal parasites, possibly because of inexperience or more risk-taking behavior. Giardiasis occured more often in long-term travelers to sub-Saharan Africa than in short-term travelers there (OR 1.93, p<.0001); that difference was not apparent for travelers to South America and Southeast Asia.

Epidemiologic surveillance of PCVs (1985–1987, >5,500 volunteers) found similar results: among the most common illnesses during service were diarrhea and giardiasis (*16*). More recently, the major health problems experienced by PCVs in Madagascar were gastrointestinal, dermatologic, and respiratory (*5*). Examination during home leave of British missionaries who served in 27 countries found diarrhea and giardiasis to be the most common problems, and those who served in West Africa had more illnesses (*7*). Not surprisingly, children of missionaries encountered poor water treatment and food sanitation (*2*); before hepatitis A vaccine was available, a questionnaire of mission boards identified viral hepatitis as the most serious health problem among missionaries (*4*). Among 328 North American missionaries evaluated during 1967–1984, 5.8% seroconverted to hepatitis A (this percentage may underestimate risk without prophylaxis because they presumably had received immune globulin); 0% seroconverted to hepatitis E after an average of 7.3 years of service (*17*). We found a higher risk for hepatitis A in long-term travelers, but the difference was not statistically significant (OR 1.21, p = 0.5328). With the wide availability of hepatitis A vaccine today and the consensus for its broad use for travel to developing regions, most travelers, especially those planning long-term travel, are expected to have been vaccinated.

A major vaccine-preventable disease is typhoid fever. Long-term travelers more frequently had enteric fever (typhoid and paratyphoid) than did short-term travelers (9/1,000 vs. 5/1,000 travelers; OR 1.70, p = 0.0024). A past estimate of the attack rate for typhoid in expatriates was 3/100,000 travelers per month of stay (*18*). Enteric fever was significantly associated with travel to south-central Asia, reflecting the distribution of enteric fever; vaccination should particularly be emphasized to long-term travelers, even though the efficacy of currently available vaccines is only 60%–70%.

A survey of corporate expatriates found that food safety practices worsened as duration of stay increased (*3*). Adherence to food and water precautions is difficult to maintain, as noted in a survey of 140 travelers in India whose median trip duration was 5 months (*19*). None had adhered fully to food and water precautions; 83% had diarrhea, and 60% had diarrhea for ≈3% of their journey time.

### Vector Transmission

Long-term travelers more frequently had vector-borne diseases than did short-term travelers because of the longer period during which bites can occur and possibly less vigilance about personal protection measures and/or chemoprophylaxis during long stays. Long-term travelers also may have more primitive, remote, and rural living conditions than short-term travelers. Leishmaniasis, malaria, and filariasis were all reported more frequently in long-term travelers than in short-term travelers (14, 68, and 5/1,000 vs. 3, 39, and 2/1,000, respectively; p<0.0001). Regional variations were consistent with geographic disease distribution (Table 1; Appendix Table 3). Other associations of long-term travel and illness were male gender (leishmaniasis, malaria), VFR (malaria, filariasis), and missionary/volunteer/aid work/research (filariasis). Posttravel medical evaluation of 212 British missionaries indicated malaria as among the most common overseas illnesses (87.3/1,000 person-years at risk); more illnesses were associated with west Africa (688/1,000 person years at risk) than other regions (*7*). Among PCVs in Madagascar, 11 (15.9%) had malaria (8 cases/100 PCV-years) (*5*). Children of missionaries received suboptimal malaria prophylaxis (*2*). Business travelers, despite understanding their risk for malaria, failed to use appropriate personal protection when duration of travel increased (*20*). Corporate expatriates also adhered poorly to malaria chemoprophylaxis with longer stays in risk areas (*3*). Long-term travelers need better preparation for preventing, diagnosing, and treating malaria; novel approaches such as provision of rapid malaria tests and adequate self-treatment medication should be considered for this high-risk population. Widespread proliferation of counterfeit drugs requires long-term travelers to take adequate supplies with them (*21**,**22*).

Seroprevalence studies confirm exposures to dengue virus in regions to which it is endemic: a serosurvey of 323 development workers and family members had increased seropositivity with longer stay (*23*). Seroconversion occurred in 6.7% of 104 Israeli travelers with trips >3 months’ duration in dengue-endemic countries and 2.9% of 477 Dutch travelers to Asia (≈30/1,000 person-months of stay) (*24*,*25*). We found dengue was diagnosed less commonly in long-term travelers (OR 0.69, p = 0.0022) than in short-term travelers, perhaps because dengue has a short incubation period and many infections occurring during prolonged stays are not confirmed; diagnostic tests are usually not performed in countries endemic for dengue because of expense or lack of diagnostic capabilities.

### Psychological Diagnoses

Some psychological diagnoses were reported significantly more often in long-term travelers (OR 2.80, p<0.0001), particularly depression (OR 3.03, p<0.0001), nonmefloquine psychosis (OR 3.89, p = 0.0006), stress (OR 5.70, p<0.0001), and fatigue (OR 3.09, p<0.0001); rates of anxiety, insomnia, substance abuse, and posttraumatic stress disorder were equivalent to or lower than rates in short-term travelers. The increased number of missionary/volunteer/research/aid workers with stress was most significant (OR 32.18, (p<0.0001). Psychological rates were highest in eastern Europe and northern Africa and lowest in the Caribbean and Southeast Asia. Mission boards consider psychological conditions to be among the most common and serious conditions, specifically depression, stress, and burnout (*4*). Furthermore, psychiatric illness caused 60% of premature repatriations among British missionaries or their family members serving overseas (*7*). Nine (14%) of 66 fatalities among PCVs from 1984 to 2003 were caused by mental illness (*26*).

In a survey of 1,340 long-term travelers from Israel (mean stay 5.3 months), 151 (11.3%) had neuropsychiatric problems during travel with a higher proportion of women (54.6%) and an association with mefloquine use (*27*). Further assessment found a mean stay abroad of 5.3 months. However, data on PCVs found that mefloquine adverse events usually occurred early in prophylaxis (*28*).

Although our analysis identified no substance abuse, an earlier study of 18–30-year-old travelers to the tropics found that approximately one third of survey responders used illicit drugs during their trip, especially travelers to the Far East. The strongest predictors of drug abuse were the combination of female sex and travel to Asia, education <12 school years, age <25 years, and lack of malaria prophylaxis. Providing antidrug brochures did not affect the drug abuse rate (*29*).

### Contact or Person-to-Person Transmission

Latent tuberculosis (positive purified protein derivative [PPD] or interferon-gamma release assays) was diagnosed more commonly in long-term than in short-term travelers (11 vs. 4/1,000; OR 3.26, p<0.0001), whereas influenza was diagnosed less commonly in long-term travelers (5 vs. 8/1,000, OR 0.60, p = 0.0183). PCVs serving in Madagascar most commonly reported respiratory problems and gastrointestinal and skin conditions, including 5.8% with Mantoux of >5 mm induration (3 cases/100 volunteer-years) (*5*). Peace Corps data from January 1, 1996, through December 31, 2005, showed rates of positive PPD conversions and active TB cases to be 1.283 and 0.057 per 1,000 volunteer-months, respectively; the African region had the highest PPD conversion rate, followed by the European region (*30*). Other studies on PPD conversion among travelers have reported rates up to 3.5/1,000 person-months (*31*).

Acute mononucleosis syndromes was significantly higher in long-term than in short-term travelers (11 vs. 7/1,000 travelers, OR 1.60, p = 0.0024) but no significant difference for other diseases transmitted through sex or body fluids. Younger age, male sex, lack of pretravel advice, and exposure in western Europe were associated with diagnoses of acute mononucleosis syndromes.

Among PCVs in Madagascar, the reported incidence of sexually transmitted infections was 6.9% (5.6% of females and 13.3% of males); 8.7% of those volunteers needed postexposure prophylaxis for human immunodeficiency virus (*5*). Long-term missionaries in developing countries had seroconversion rates of 5.5% to antibody to hepatitis B core antigen and 0.6% to antibody to hepatitis C virus, suggesting significant exposure to hepatitis B (*17*). In our study, hepatitis B infection was diagnosed more often in short-term than long-term travelers, although not significantly so (OR 0.67, p = 0.3131). More potential exposures to hepatitis B is expected during long-term travel; therefore, hepatitis B vaccine is routinely recommended for long-term travelers. (*32*). A plausible explanation for our result is that a high percentage of long-term travelers had been vaccinated against hepatitis B. Hepatitis B infection, a vaccine-preventable disease, should be targeted for prevention in travelers.

### Environmental Cause

Among diagnoses attributed to soil and water exposure, schistosomiasis and strongyloidiasis were more common in long-term travelers (24 and 7/1,000 travelers; OR 4.26, p<0.0001, and 1.85, p = 0.0021, respectively) and were associated with males. Schistosomiasis was also associated with tourism and missionary/volunteer/research/aid work, and strongyloidiasis was associated with VFR. An earlier study found that 8 (11.6%) of 69 PCVs who served in Madagascar had antischistosomal antibodies (*5*).

Although ectoparasites (scabies, sand flea, and head lice) were reported in 11.6% of PCVs (*5*), long-term travelers in our study, compared with short-term travelers, had a proportionately lower number of other skin conditions with possible environmental exposures (OR 0.29, p<0.0001) but a proportionately higher number of fungal skin infections (OR 2.33, p<0.0001).

Rabies risk has been considered to increase with longer duration of travel, and preexposure prophylaxis is typically recommended, particularly for long stays. Our data showed a lower proportionate need for postexposure prophylaxis (not actual rabies) in long-term travelers than in short-term travelers (7 vs. 23/1,000, OR 0.31, p<0.0001), possibly because long-term travelers may be more knowledgeable or better educated about animal exposure risks, more likely to avoid exposures, and more likely to have been vaccinated before travel. In PCVs, potential exposure to rabies was 10× higher abroad than domestically (*1*). Among Norwegian missionaries serving in developing countries for 4–5 years, 7% had possible rabies exposure (*33*). A study of travelers returning from travel >1 month identified 1.6% injured by a potentially rabid animal (mainly dog and monkey), or 2.66 per 1,000 travelers per month (*34*). Those injured had significantly longer trips (mean 6.9 mo, ±3.8 SD); only 31% sought appropriate medical treatment (*34*).

### Injury

Mission boards consider injury among the most common and serious conditions (*4**,**35*). The leading cause of death in Africa for American missionaries during 1970–1985 was motor vehicle injury (*35*). Of 66 deaths occurring in PCVs from 1984 to 2003, injury was the primary cause (45), including motor vehicle injuries, most commonly by automobile but also by bus, truck, taxi, minibus taxi, and motorcycle (*26*). Among 1,190 returned expatriates, most of whom had served the International Committee of the Red Cross for >6 months, 10% reported injury or accident during their service (*36*). Injured travelers are unlikely to seek care at a GeoSentinel site, so our analysis has limited data about injury.

### Limitations

Our findings are subject to several limitations. The GeoSentinel database captures travelers who sought treatment at specialized travel and tropical medicine clinics and who may not be representative of all travelers. Long-term travelers may be more likely than short-term travelers to seek a GeoSentinel site because of concern about unusual tropical diseases. We used only GeoSentinel data, so proportionate frequency of diagnoses for long-term travelers compared with short-term travelers in this database can be derived, but not risk for illness. Missing travel duration eliminated ≈10% of records from analysis. Paucity of injury data is another limitation of the analysis because injury is a major cause of illness and death in long-term travelers. Similarly, travelers with dental, ophthalmologic, obstetric, and gynecologic problems rarely visit GeoSentinel sites.

## Conclusions

Approximately 10% of all ill travelers seen at GeoSentinel sites are long-term travelers. Long-term travelers are characterized by male gender and travel for missionary/volunteer/research or business; most had pretravel evaluations. Among the problems more frequently seen in long-term travelers than in short-term travelers are infections with long incubation and long-lasting or chronic durations; malaria is especially important, as are leishmaniasis, filariasis, gastrointestinal parasites, schistosomiasis, and latent tuberculosis. Many vector-borne diseases with short incubation periods (e.g., dengue, chikungunya, rickettsia) are diagnosed more often in short-term travelers. These findings do not mean that these infections occur less often in long-term travelers, only that they are not active when long-term travelers are seen after travel; a similar situation may be true for animal bites. Many common infections seen in long-term travelers are preventable by vaccines, vector avoidance, food/water precautions, and avoidance of soil and fresh water. Psychological problems, especially depression, stress, nonmefloquine psychosis, and prolonged fatigue, increase with long-term travel. The high OR (32.18) of missionary/volunteer/research/aid workers with stress merits attention and intervention. Clinicians must be alert to psychological problems and manage them, as reentry and readjustment for long-term travelers may be difficult. Among the vaccine-preventable diseases, enteric fever and hepatitis A increase with long-term travel. Because >50% of ailments for which long-term travelers visit a healthcare provider are preventable and 70% of long-term travelers had pretravel visits, opportunities exist to educate, vaccinate, provide malaria chemoprophylaxis, and prepare these travelers for possible break-through infection.

Disease patterns differed significantly for long-term and short-term travelers. Particular areas of concern for long-term travelers are vector-borne, ingestion-transmitted, contact-transmitted disease, and psychological problems. Our results can help identify priorities for screening and diagnosing illnesses in long-term travelers and for providing evidence-based pretravel advice.

## Supplementary Material

Appendix Table 1 GeoSentinel Surveillance Network demographics of long-term and short-term travelers (N = 28,846), June 1996-December 2008*

Appendix Table 2 Frequency of diagnoses by syndrome groups in long-term and short-term travelers (N = 33,360), GeoSentinel Surveillance Network, June 1996-December 2008*

Appendix Table 3 Summary table of logistic regression performed on long-term travelers seen after travel showing significant variables associated with common diagnoses, GeoSentinel Surveillance Network, June 1996-December 2008*
