# Supplementary material for: Illness in Long-Term Travelers Visiting GeoSentinel Clinics
Source: Emerg Infect Dis. 2009 Nov;15(11):1773–82. doi: 10.3201/eid1511.090945 (PMC2857257; doi:10.3201/eid1511.090945)
Supplement: Appendix Table 1 — GeoSentinel Surveillance Network demographics of long-term and short-term travelers (N = 28,846), June 1996-December 2008* [file 09-0945_appT1-s1.pdf]

Publisher: CDC; Journal: Emerging Infectious Diseases
Article Type: Research; Volume: 15; Issue: 11; Year: 2009; Article ID: 09-0945
DOI: 10.3201/eid1511.090945; TOC Head: Research

Appendix Table 1. GeoSentinel Surveillance Network demographics of long-term and short-term travelers (N = 28,846), June 1996–December 2008*

Characteristic	Travel <1 mo, % travelers, n = 24,807	Travel >6 mo, % travelers, n = 4,039
Age†		
<20	7‡	7‡
20–64	88	90
≥65	5	3
Sex		
M	50	57
F	50	43
Reason for travel		
Business	14	26
Missionary/volunteer/research/aid work	7	39
Student	1	2
Tourism	71	29
VFR	8	4
Patient type		
Inpatient	9§	9§
Outpatient	89	90
Teleconsult, inpatient	1	1
Teleconsult, outpatient	1	0
Pretravel encounter		
Yes	49	70
No	39	18
Unknown	12	12
Interval between return from travel to seeking care, wk		
≤1	36	32
1–6	37	38
≥6	27	30
Country or region of origin		
Australia/New Zealand	3	6
Caribbean	1	0
Eastern Europe	2	1
Middle East	4	12
North Africa	1	0
North America	22	29
Northeast Asia	5	2
South America	2	1
South-central Asia	2	1
Southeast Asia	2	2
Sub-Saharan Africa	4	3
Western Europe	52	43

Risk qualifier		
Prearranged	46	18
Risk travel	45	28
Expatriate	9	54
Region of exposure		
Antarctica	0	0
Australia/New Zealand	0	1
Caribbean	8	2
Central America	8	7
Eastern Europe	1	1
Middle East	2	2
North Africa	6	3
North America	1	0
Northeast Asia	3	3
Oceania	1	2
South America	7	16
South-central Asia	13	14
Southeast Asia	19	13
Sub-Saharan Africa	25	34
Western Europe	5	1

*Mean trip duration for long-term travelers was 697 d. VFR, visiting friends and relatives.
†Mean age: short-term, 38; long-term, 33.
‡Rounded to whole numbers: travel <1 month, 6.93; travel >6 months, 7.10.
§Rounded to whole numbers: travel <1 month, 9.09; travel >6 months, 9.04.
