# Supplementary material for: Illness in Long-Term Travelers Visiting GeoSentinel Clinics
Source: Emerg Infect Dis. 2009 Nov;15(11):1773–82. doi: 10.3201/eid1511.090945 (PMC2857257; doi:10.3201/eid1511.090945)
Supplement: Appendix Table 2 — Frequency of diagnoses by syndrome groups in long-term and short-term travelers (N = 33,360), GeoSentinel Surveillance Network, June 1996-December 2008* [file 09-0945_appT2-s2.pdf]

Publisher: CDC; Journal: Emerging Infectious Diseases
Article Type: Research; Volume: 15; Issue: 11; Year: 2009; Article ID: 09-0945
DOI: 10.3201/eid1511.090945; TOC Head: Research

Appendix Table 2. Frequency of diagnoses by syndrome groups in long-term and short-term travelers (N = 33,360), GeoSentinel Surveillance Network, June 1996–December 2008*

			Syndromes diagnosed after travel /1,000 travelers with exposure in each region										
Major syndromes	<1 mo, n = 28,618	>6 mo, n = 4,742	Caribbean, n = 99	Central America, n = 269	Eastern Europe, n = 51	Middle East, n = 64	North Africa, n = 135	Northeast Asia, n = 114	Oceania, n = 91	South America, n = 654	South-central Asia, n = 573	Southeast Asia, n = 523	Sub-Saharan Africa, n = 1,409
Febrile/ systemic illness	191	154	141	108	118	63	104	61	330	93	155	224	214
Acute diarrhea	221	133	81	149	177	234	141	114	55	125	243	115	107
Dermatologic	180	118	202	97	20	94	74	140	110	217	94	128	88
Chronic diarrhea	72	94	71	208	177	31	52	79	11	133	133	86	53
Gastrointestinal, other	53	77	71	67	39	47	59	123	33	83	84	59	83
Nonspecific symptoms or findings	30	52	30	56	59	31	44	44	55	51	70	44	50
Respiratory	83	38	51	22	78	31	22	53	55	18	38	46	41
Chronic disease	13	23	40	19	39	47	15	35	22	15	18	46	16
Psychological	10	22	10	22	39		37	18		17	16	10	19
Miscellaneous tissue parasites	6	20							22	8	5	12	42
Genitourinary and STIs	22	18	20	11		16	22	9		8	7	29	28
Injury and musculoskeletal	19	16	51	7	20		15		11	17	18	13	15
Neurologic	16	14	10	37		16	22	9		9	5	17	14

*Long term, travel >6 months' duration; short term, travel <1 month's duration. STIs, sexually transmitted infections.
