# Supplementary material for: Illness in Long-Term Travelers Visiting GeoSentinel Clinics
Source: Emerg Infect Dis. 2009 Nov;15(11):1773–82. doi: 10.3201/eid1511.090945 (PMC2857257; doi:10.3201/eid1511.090945)
Supplement: Appendix Table 3 — Summary table of logistic regression performed on long-term travelers seen after travel showing significant variables associated with common diagnoses, GeoSentinel Surveillance Network, June 1996-December 2008* [file 09-0945_appT3-s3.pdf]

Publisher: CDC; Journal: Emerging Infectious Diseases
Article Type: Research; Volume: 15; Issue: 11; Year: 2009; Article ID: 09-0945
DOI: 10.3201/eid1511.090945; TOC Head: Research

Appendix Table 3. Summary table of logistic regression performed on long-term travelers seen after travel showing significant variables associated with common diagnoses, GeoSentinel Surveillance Network, June 1996–December 2008*

Diagnosis	Associated variables	Odds ratio (p value)
Leishmaniasis	Male sex	3.45 (0.0001)
	Travel to North Africa	26.42
	Travel to South America	49.99
	Travel to South-central Asia	7.29 (0.0003)
	Business	0.11 (0.0003)
	Missionary/volunteer/aid/research	0.05
Malaria	Male sex	1.95
	Travel to Southeast Asia	3.68
	Travel to Oceania	23.86
	Travel to sub-Saharan Africa	8.44
	Missionary/volunteer/aid/research	0.57
	VFR	1.79 (0.0021)
Chronic diarrhea	Pretravel advice	1.64 (0.0008)
	Travel to South-central Asia	1.72 (0.0009)
	Travel to Central America	2.62
	Tourism	2.14
Giardiasis	Age	0.99 (0.0009)
	Travel to Middle East	3.27 (0.0068)
	Travel to South-central Asia	1.87
Gastrointestinal parasite	Age	0.99 (0.0004)
	Travel to South-central Asia	2.04
	Travel to Oceania	0.21 (0.0271)
	Travel to Caribbean	0.29 (0.0372)
Enteric fever	Travel to South-central Asia	9.35
Latent tuberculosis	Travel to Southeast Asia	2.95 (0.0012)
	Tourism	0.03
	Business	0.23 (0.0002)
	Missionary/volunteer/aid/research	0.41 (0.0089)
Acute mononucleosis syndrome	Age	0.95 (0.0009)
	Travel to Southeast Asia	3.74 (0.0016)
	Travel to Western Europe	9.81 (0.0030)
	Travel to South America	3.83
	Travel to Central America	5.21
	Missionary/volunteer/aid/research	5.52
Schistosomiasis	Male sex	1.58 (0.0199)
	Travel to sub-Saharan Africa	4.86
	Travel to South America	0.16 (0.0122)
	Travel to South-central Asia	0.18 (0.0051)
	Tourism	7.80

	Missionary/volunteer/aid/research	5.15
Strongyloides	VFR	6.75
Superficial cutaneous mycosis	Male sex	2.24 (0.0041)
	Travel to Western Europe	4.94 (0.0309)
	Pretravel advice	1.99 (0.0252)
Depression	Travel to South-central Asia	0.24 (0.0196)
	Tourism	0.21 (0.0107)
Psychosis nonmefloquine	Travel to Eastern Europe	32.39 (0.0020)
	Tourism	4.94 (0.0310)
Stress	Age*	1.02 (0.0013)
	Female sex	2.23 (0.0265)
	Travel to North Africa	3.12 (0.0125)
	Travel to South-central Asia	0.33 (0.0339)
	Missionary/volunteer/aid/research	32.18
Fatigue	Pretravel advice	1.93 (0.0111)
	Travel to Western Europe	4.77 (0.0116)
	Travel to South-central Asia	2.46 (0.0005)
	Travel to Central America	2.14 (0.0487)
	Tourism	1.76 (0.0105)

*Long term, travel >6 months' duration; VFR, visiting friends and relatives. Age was a continuous variable. For each 1-year change in age, the likelihood of having giardiasis changes by 0.99 (in this case, a decrease).
†p<0.0001 unless specified.
